# Heat-Processed *Scutellariae* Radix Enhances Anti-Inflammatory Effect against Lipopolysaccharide-Induced Acute Lung Injury in Mice via NF-***κ***B Signaling

**DOI:** 10.1155/2015/456846

**Published:** 2015-06-18

**Authors:** Yu Ock Shin, Chan Hum Park, Gyeong-Hwan Lee, Takako Yokozawa, Seong-Soo Roh, Man Hee Rhee

**Affiliations:** ^1^College of Veterinary Medicine, Kyungpook National University, Daegu 702-701, Republic of Korea; ^2^College of Korean Medicine, Daegu Haany University, Daegu 706-060, Republic of Korea; ^3^Jeollanamdo Development Institute for Korean Traditional Medicine, Jeollanamdo 529-851, Republic of Korea; ^4^Molecular Inflammation Research Center for Aging Intervention, Pusan National University, Busan 609-735, Republic of Korea; ^5^Graduate School of Science and Engineering for Research, University of Toyama, Toyama 930-8555, Japan

## Abstract

The present study was conducted to examine whether heat-processed* Scutellariae* Radix has an ameliorative effect on lipopolysaccharide- (LPS-) induced acute lung injury in mice. The effects of* Scutellariae* Radix heat-processed at 160°C (HSR) were compared with those of nonheat-processed* Scutellariae* Radix (NSR). The LPS-treated group displayed a markedly decreased body weight and significantly increased lung weight; however, the administration of NSR or HSR improved both the body and lung weights. The increased oxidative stress and inflammatory biomarker levels in the serum and lung were reduced significantly with HSR. The reduced superoxide dismutase and catalase increased significantly by both NSR and HSR. Also, the dysregulated oxidative stress and inflammation were significantly ameliorated by NSR and HSR. The expression of inflammatory mediators and cytokines by nuclear factor-kappa B activation was modulated through inhibition of a nuclear factor kappa B*α* degradation. Also, lung histological change was markedly suppressed by HSR rather than NSR. Overall, the ameliorative effects of HSR were superior to those when being nonheat-processed. The representative flavonoid contents of* Scutellariae* Radix that include baicalin, baicalein, and wogonin were greater by heat process. These data reveal heat-processed* Scutellariae* Radix may be a critical factor involved in the improvement of lung disorders caused by LPS.

## 1. Introduction

Acute lung injury is a form of acute respiratory failure, which is characterized by increased pulmonary vascular permeability, pulmonary edema, excessive neutrophil migration, and the release of proinflammatory cytokines and mediators [1, 2]. Thus, it leads to marked morbidity and mortality [3]. The etiologies of acute lung injury are severe hypoxemia, bilateral infiltration of leukocytes, pulmonary edema, pneumonia, and trauma. In particular, the incidence of acute lung injury in the US, with 200,000 newly diagnosed cases per year, is very high [1]. Therefore, highly effective drugs and therapies are required for the treatment of acute lung injury.

Lipopolysaccharide (LPS), the major component of the outer membrane of Gram-negative bacteria, has been generally used in an experimental lung injury model [4, 5]. LPS has been reported to activate macrophages to produce proinflammatory cytokines and mediators. Also, it promotes the production of reactive oxygen species (ROS), such as superoxide (O_2_^−^), hydrogen peroxide (H_2_O_2_), and hydroxyl radicals in macrophages [6]. Nitric oxide (NO) induced after LPS challenge is a potent inflammatory mediator that reacts with O_2_^−^ and produces peroxynitrite (ONOO^−^). ONOO^−^ is linked to cell death and lung injury. The excessive production of ROS suppresses the innate antioxidant system. So, the oxidant/antioxidant systems become unbalanced [7].

*Scutellariae* Radix (*Scutellaria baicalensis* Georgi) has been used to treat high fever, diarrhea, jaundice, hypertension, and bacterial and viral infections as herbal medicine in China, Japan, and Korea. Traditionally, herbal medicine is comprised of a complex mixture of biologically active components, and some undergo heat processing such as air drying, steaming, roasting, and baking to enhance the therapeutic efficacy [8]. These processing methods increase their biological activity through the chemical alteration of components [9, 10] and also improve the bioactive gredients through increasing the total polyphenol contents, including flavonoids [11, 12]. However,* Scutellariae* Radix has yet to be reported regarding its chemical profiling and biological activity following heat-processing. Therefore, we measured the contents of three flavonoids, baicalin, baicalein, and wogonin, of nonheat-processed and heat-processed* Scutellariae* Radix and evaluated the anti-inflammatory activities of these forms of* Scutellariae* Radix on LPS-induced acute lung injury in mice.

## 2. Materials and Methods

### 2.1. Materials

LPS from* Escherichia coli* serotype 0127:B8 (purity > 99%; made up of a hydrophobic lipid (lipid A, which is responsible for the toxic properties of the molecule), a hydrophilic core polysaccharide chain, and a hydrophilic O-antigenic polysaccharide side chain), phenylmethylsulfonyl fluoride (PMSF), and dithiothreitol (DTT) were purchased from Sigma Aldrich Co., Ltd. (St. Louis, MO, USA). The protease inhibitor mixture and ethylenediaminetetraacetic acid (EDTA) were purchased from Wako Pure Chemical Industries, Ltd. (Osaka, Japan). 2′,7′-Dichlorofluorescein diacetate (DCFH-DA) was obtained from Molecular Probes (Eugene, OR, USA). ECL Western Blotting Detection Reagents and pure nitrocellulose membranes were purchased from GE Healthcare (Buckinghamshire, UK). Nuclear factor-kappa B (NF-*κ*B)p65, inhibitor of nuclear factor kappa B (I*κ*B)*α*, I*κ*B*β*, mouse monoclonal antibody cyclooxygenase-2 (COX-2), inducible nitric oxide synthase (iNOS), monocyte chemotactic protein-1 (MCP-1), intracellular adhesion molecule-1 (ICAM-1), tumor necrosis factor *α*(TNF-*α*), interleukin-6 (IL-6), superoxide dismutase (SOD), catalase, *β*-actin, histone, and goat anti-rabbit and goat anti-mouse IgG horseradish peroxidase- (HRP-) conjugated secondary antibodies were purchased from Santa Cruz Biotechnology, Inc. (Santa Cruz, CA, USA). The solvents, such as acetonitrile, methanol, formic acid, and water, were of high purity high-performance liquid chromatography (HPLC) grade and obtained from Merck (Darmstadt, Germany). The standard materials of baicalin, baicalein, and wogonin for HPLC were purchased from Wako Pure Chemical Industries, Ltd. (Osaka, Japan).

### 2.2. Plant Materials and Their Heat Processing

*Scutellariae* Radix was purchased from Ominherb Co. (Youngcheon, Korea). A voucher herbarium specimen has been deposited at the Herbarium of Daegu Haany University and was identified by Professor S.S. Roh, the herbarium leader of Daegu Haany University. Dried slices of* Scutellariae* Radix were heat-processed at 160°C (internal temperature) for 7 min using a roasting machine (Genesis Co., Ltd., Kyungki-do, Korea). The material was pulverized and extracted by 60-Hz ultrasonic waves at room temperature for 3 h, and the solvent was evaporated* in vacuo* to give an extract with a yield of 22.4%, by weight, of the original* Scutellariae* Radix. Nonheat-processed material was also pulverized and extracted by the same method as described above, giving an extract with a yield of 22.8%.

### 2.3. Analysis of Baicalin, Baicalein, and Wogonin

**Figure 1:**
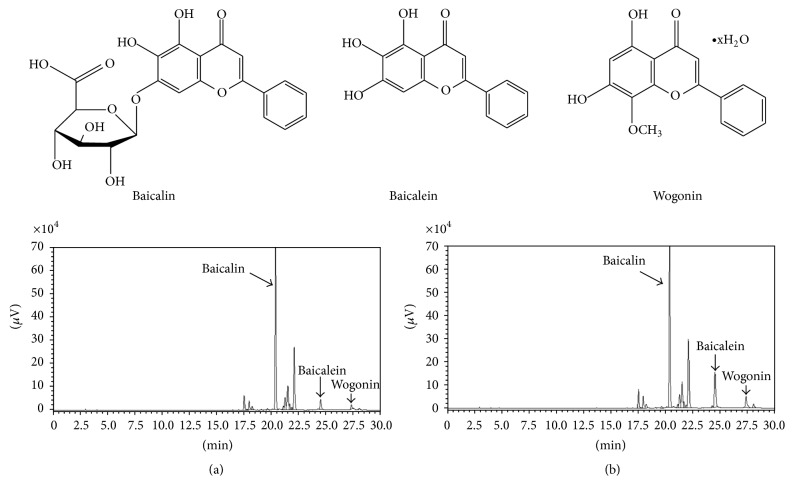
HPLC profile of nonheat-processed (a) and heat-processed (b)* Scutellariae* Radix.

The 70% ethanol extract of each sample (10 mg) was dissolved in 10 mL of 50% methanol with multivortexing, and filtered through a Dismic-13 JP membrane filter (Advantec Toyo, Tokyo, Japan; pore diameter: 0.2 *μ*m). We injected 20 *μ*L of the sample into a reverse-phase HPLC using a Phenomex Gemini NX C18 (4.6 × 150 mm, 3-*μ*m pore size), with a column temperature of 35°C. Mobile phase component A = 0.1% formic acid (aq.) and B = acetonitrile. The gradient conditions were as follows: 0 min, 0% B; 3 min, 0% B; 5 min, 10% B; 7 min, 10% B; 12 min, 20% B; 17 min, 30% B; 22 min, 30% B; 31 min, 60% B; 35 min, 60% B; 40 min, 95% B; 43 min, 95% B; 45 min, 50% B. The flow rate was 0.6 mL/min. The UV absorbance from 277 nm was monitored using an Agilent 1200 series with a multiwavelength detector (Agilent Technologies, San Jose, CA, USA). All peaks were assigned by carrying out coinjection tests with authentic samples and comparing them with the UV spectral data. The components of major compounds (baicalin, baicalein, and wogonin) were detected from nonheat-processed* Scutellariae* Radix and 160°C heat-processed* Scutellariae* Radix extracts. The measurement was repeated three times for each sample. Representative HPLC results are illustrated in Figure 1. The amount of each flavonoid was as follows: nonheat-processed* Scutellariae* Radix: 146.7 mg/g baicalin, 11.1 mg/g baicalein, 1.7 mg/g wogonin; 160°C heat-processed* Scutellariae* Radix: 154.5 mg/g baicalin, 38.6 mg/g baicalein, 6.1 mg/g wogonin.

### 2.4. Experimental Animals and Treatment

Experiments were performed according to the “Guidelines for Animal Experimentation” approved by Daegu Haany University. Six-week-old male ICR mice were purchased from Samtako (Osan, Korea). Losonczy et al. [13] reported that male mice may be sensitized to LPS-induced shock and that the sensitivity of males to endotoxin is associated with an attenuated, not exaggerated, and total rate of NO synthesis. So, this experiment was conducted using male mice. The animals were maintained under a 12 h light/dark cycle and housed with a controlled temperature (24°C) and humidity (55 ± 5%). After adaptation (1 week), the mice were divided into four groups of equal number (*n* = 8, each), avoiding any intergroup differences in body weight. The normal and vehicle-treated LPS groups were given water using a stomach tube, while the other groups were orally administered nonheat-processed* Scutellariae* Radix or heat-processed* Scutellariae* Radix at a dose of 100 mg/kg body weight daily using a stomach tube for 3 consecutive days. The mice were given intraperitoneal LPS at 20 mg/kg body weight. At 24 h after LPS challenge, blood samples were collected by cardiac puncture from anesthetized mice. The serum was immediately separated from the blood samples by centrifugation. Subsequently, the lung was perfused through the artery with ice-cold physiological saline (0.9% NaCl, pH 7.4), removed, quickly frozen, and kept at −80°C until analysis.

### 2.5. Measurement of Immune Response-Associated Secreted Factors in the Serum

Proinflammatory biomarkers in the serum, including MCP-1 and IL-6, and regulated on activation normal T cells expressed and secreted (RANTES), were assayed using the multiplexed bead-based immunoassay Milliplex Map, MPXMCYTO70KPMX32 (Millipore, Billerica, MA, USA).

### 2.6. Measurement of Nitrite (NO_2_^−^) and Nitrate (NO_3_^−^) Levels in the Serum

NO_2_^−^ and NO_3_^−^ levels were measured primarily following the method of Misko et al. [14]. Briefly, serum was filtered through an Ultrafree-MC microcentrifuge filter unit (Millipore, Bedford, MA, USA) for 1 h at 14,000 rpm to remove hemoglobin released by cell lysis. As NO_2_^−^ in serum is mostly oxidized to NO_3_^−^ by reaction with the iron-heme center of hemoglobin, the resulting NO_3_^−^ was first reduced to NO_2_^−^ by incubation with nitrate reductase and measured by a microplate assay method based on the Griess reaction [15].

### 2.7. Measurement of ONOO^−^ Level in the Serum

The ONOO^−^ level was evaluated using the method of Kooy et al. [16] with minor modifications. Serum was added to the rhodamine solution [50 mM sodium phosphate buffer, 90 mM sodium chloride, 5 mM diethylenetriaminepentaacetic acid, and dihydrorhodamine (DHR) 123], and then the fluorescence of rhodamine 123, the reduced form of DHR 123, at 485 nm excitation and 535 nm emission was measured every 5 min for 30 min with a fluorescence plate reader.

### 2.8. Measurement of ROS Level in the Serum and Lung

ROS levels in the serum and lung were measured employing the method of Ali et al. [17]. Lung tissues were homogenized on ice with 1 mM EDTA-50 mM sodium phosphate buffer (pH 7.4), and then 25 mM DCFH-DA was added to homogenates or serum. After incubation for 30 min, the changes in fluorescence values were determined at an excitation wavelength of 480 nm and emission wavelength of 535 nm.

### 2.9. Assessment of NO and ONOO^−^ Generation in the Lung

NO was measured by assaying the lung tissue, using the method of Green et al. [15]. Each sample was mixed with an equal volume of Griess reagent (Promega Corporation, Madison, WI, USA) and incubated at room temperature for 10 min. Absorbance was measured at 540 nm with an ELISA reader. The nitrite level in the samples was determined by comparison against a sodium nitrite curve using the method of Misko et al. [14]. ONOO^−^ was measured by the method of Kooy et al. [16]. Each sample was mixed with rhodamine buffer (pH 7.4) and 5 mM DHR 123. After incubation for 5 min at 37°C, the fluorescence intensity of the oxidized DHR 123 was measured with a microplate fluorescence reader at excitation and emission wavelengths of 485 and 530 nm, respectively.

### 2.10. Preparation of Nuclear and Postnuclear Fractions

Nuclear protein extraction was performed according to the method of Komatsu [18]. In brief, lung tissues were homogenized with ice-cold lysis buffer containing 5 mM Tris-HCl (pH 7.5), 2 mM MgCl_2_, 15 mM CaCl_2_, and 1.5 M sucrose, and then 0.1 M DTT and protease inhibitor mixture solution were added. After centrifugation (10,500 ×g for 20 min at 4°C), the pellet was suspended with extraction buffer containing 20 mM 2-[4-(2-hydroxyethyl)-1-piperazyl] ethanesulfonic acid (pH 7.9), 1.5 mM MgCl_2_, 0.42 M NaCl, 0.2 mM EDTA, and 25% (v/v) glycerol, and then 0.1 M DTT and protease inhibitor mixture solution were added. The mixture was placed on ice for 30 min. The nuclear fraction was prepared by centrifugation at 20,500 ×g for 5 min at 4°C. The postnuclear fraction was extracted from the lung of each mouse, as described below. In brief, lung tissue was homogenized with ice-cold lysis buffer (pH 7.4) containing 137 mM NaCl, 20 mM Tris-HCl, 1% Tween 20, 10% glycerol, 1 mM PMSF, and protease inhibitor mixture solution. The homogenate was then centrifuged at 2,000 ×g for 10 min at 4°C. The protein concentration in each fraction was determined using a Bio-Rad protein kit (Bio-Rad Laboratories, Hercules, CA, USA).

### 2.11. Immunoblotting Analyses

For the determination of NF-*κ*Bp65 and histone, 10 *μ*g of protein from each nuclear fraction was electrophoresed through 12% sodium dodecylsulfate polyacrylamide gel (SDS-PAGE). Separated proteins were transferred to a nitrocellulose membrane, blocked with 5% (w/v) skim milk solution for 1 h, and then incubated with primary antibodies to NF-*κ*Bp65 and histone overnight at 4°C. After the blots were washed, they were incubated with anti-rabbit or anti-mouse IgG HRP-conjugated secondary antibody for 1 h at room temperature. Also, 10–15 *μ*g of protein of each postnuclear fraction of SOD, catalase, I*κ*B*α*, I*κ*B*β*, COX-2, iNOS, MCP-1, ICAM-1, TNF-*α*, IL-6, and *β*-actin was electrophoresed through 8–15% SDS-PAGE. Each antigen-antibody complex was visualized using ECL Western Blotting Detection Reagents and detected by chemiluminescence with Sensi-Q 2000 (Lugen sci, Gyeonggi-do, Korea). Band densities were determined using ATTO Densitograph Software (ATTO Corporation, Tokyo, Japan) and quantified as the ratio to histone or *β*-actin. The protein levels of groups are expressed relative to those of normal mice.

### 2.12. Histological Examination of Lung Tissue

For microscopic evaluation, the lung was cut to isolate the middle segment. This segment was fixed in 10% neutral-buffered formalin and after embedding in paraffin, cut into 2 *μ*m sections and stained using hematoxylin and eosin (H/E) for microscopic evaluation. The stained slices were subsequently observed under an optical microscope and analyzed using the i-Solution Lite software program (Innerview Co.).

### 2.13. Statistical Analysis

Data are expressed as means ± SEM. Significance was assessed by one-way analysis of variance (ANOVA) followed by Dunnett's multiple comparison test (SPSS 11.5.1 for Windows, 2002, SPSS Inc., USA). Values of *p* < 0.05 were considered significant.

## 3. Results

### 3.1. Changes in Body and Lung Weights

Table 1 shows the changes in body and lung weights during the experimental period. The LPS-induced acute lung injury models displayed a markedly decreased body weight, and the decreased body weight was significantly increased by both the nonheat-processed and heat-processed* Scutellariae* Radix administrations. Challenge of animals with LPS resulted in significant increases in the lung weight (207% of normal value), suggestive of pulmonary edema and infiltration. On the other hand, pretreatment with nonheat-processed or heat-processed* Scutellariae* Radix was effective for preventing LPS-induced increases in the lung weight (139 and 111% of normal value, resp.).

### 3.2. Biochemical Features of Serum

Table 2 shows the effects of* Scutellariae* Radix on general biochemical parameters of serum. The MCP-1 and IL-6 levels of the control mice were markedly increased compared to normal mice, but they were significantly decreased by the administration of heat-processed* Scutellariae* Radix. Similarly, the RANTES level in LPS-treated mice was markedly higher than in normal mice, but it was slightly decreased in* Scutellariae* Radix-administered groups. In addition, the ROS level of LPS-treated mice was significantly elevated to 72.7 fluorescence/min/mL in comparison with that of normal mice at 42.7 fluorescence/min/mL. However, it was significantly decreased in LPS-treated mice administered heat-processed* Scutellariae* Radix. The NO_2_^−^/NO_3_^−^ level was markedly increased from 15.9 *μ*mol/mL in normal mice to 191.6 *μ*mol/mL in LPS-treated control mice. It was markedly reduced by the administration of heat-processed* Scutellariae* Radix. The oral administration of nonheat-processed* Scutellariae* Radix led to no changes among the vehicle-administered and LPS-treated mice. In addition, the ONOO^−^ level was significantly decreased by both the nonheat-processed and heat-processed* Scutellariae* Radix administrations, as shown in Table 2.

### 3.3. Histological Examination of Lung Tissue

**Figure 2:**
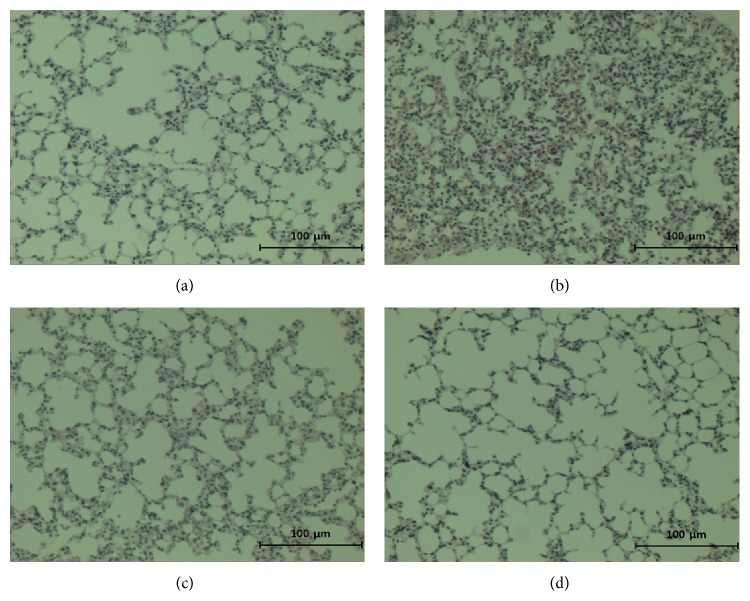
H/E staining of lung tissue. (a) Normal mice, (b) vehicle-administered and LPS-treated mice, (c) nonheat-processed* Scutellariae* Radix-administered and LPS-treated mice, and (d) heat-processed* Scutellariae* Radix-administered and LPS-treated mice. ×400.

Figure 2 shows the results of the histological examinations of lung tissue stained with H/E. Histological changes such as lung edema, an increased alveolar wall thickness, inflammatory cell aggregation, and moderate pulmonary hemorrhage were observed. The lesions of normal mice were not apparent. Moreover, pretreatment with nonheat-processed or heat-processed* Scutellariae* Radix markedly ameliorated the pulmonary injury. In particular, heat-processed* Scutellariae* Radix more effectively improved the pathological status compared with the nonheat-processed form.

### 3.4. ROS, NO, and ONOO^−^ in the Lung

Table 3 shows the effect of nonheat-processed or heat-processed* Scutellariae* Radix on the generation of ROS, NO, and ONOO^−^. The levels of ROS, oxidative stress biomarkers, in the lung of the vehicle-administered and LPS-treated mice were significantly elevated compared with normal mice. Nonheat-processed* Scutellariae* Radix administration showed a tendency to decrease the ROS levels in the lung (without significance), but those levels in the lung on receiving heat-processed* Scutellariae* Radix were significantly decreased. The NO and ONOO^−^ levels in the vehicle-administered and LPS-treated mice were elevated compared with the normal group (1.95- and 2.14-fold, resp.), whereas these levels were significantly decreased with nonheat-processed and heat-processed* Scutellariae* Radix; especially, NO was markedly reduced with the heat-processed form.

### 3.5. Antioxidant Enzyme-Related Protein Expressions in the Lung

**Figure 3:**
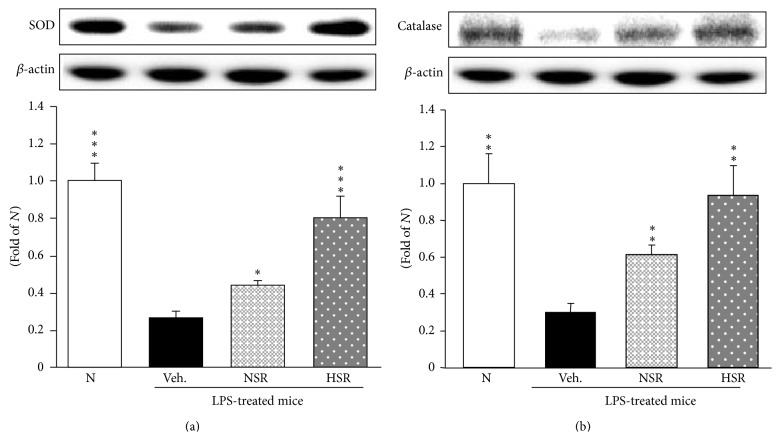
SOD (a) and catalase (b) protein expressions in lung tissue. N, normal mice; Veh., vehicle-administered and LPS-treated mice; NSR, nonheat-processed* Scutellariae* Radix-administered and LPS-treated mice; HSR, heat-processed* Scutellariae* Radix-administered and LPS-treated mice. Data are the mean ± SEM. Significance: ^*∗*^ *p* < 0.05, ^*∗∗*^ *p* < 0.01, and ^*∗∗∗*^ *p* < 0.001 versus vehicle-administered and LPS-treated mice.

To investigate SOD and catalase protein expressions in the lung, they were examined by Western blot analyses. The protein expressions of both proteins were decreased in vehicle-administered and LPS-treated mice compared with normal mice; however, nonheat-processed and heat-processed* Scutellariae* Radix administrations led to upregulations of SOD and catalase expressions (Figure 3). In particular, SOD and catalase protein expressions on heat-processed* Scutellariae* Radix administration were enhanced to nearly normal levels.

### 3.6. Oxidative Stress-Related Protein Expressions in the Lung

**Figure 4:**
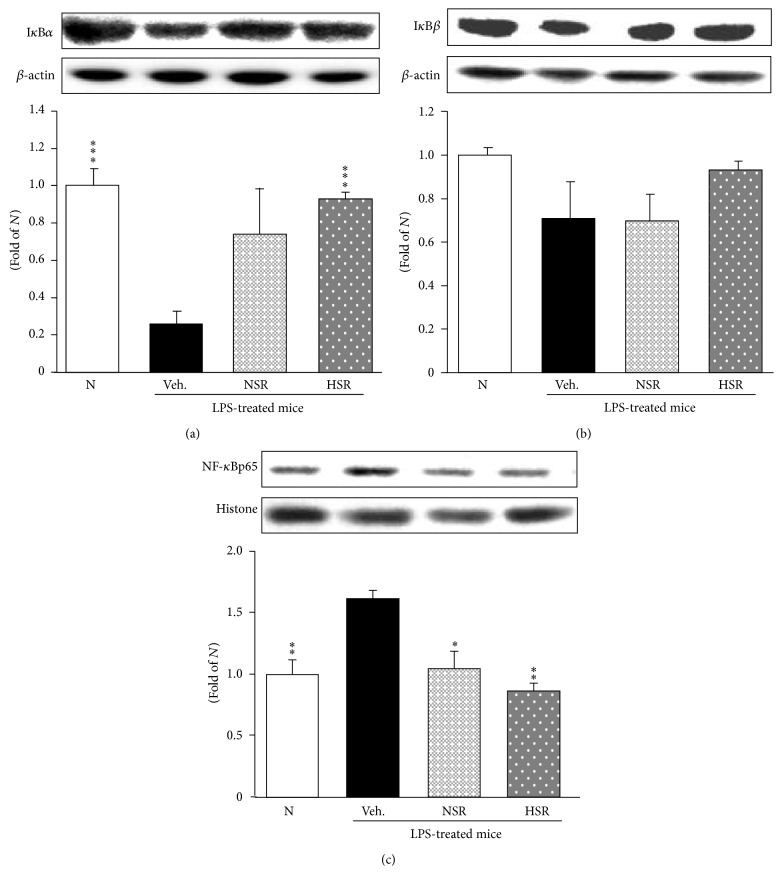
I*κ*B*α* (a), I*κ*B*β* (b), and NF-*κ*Bp65 (c) protein expressions in lung tissue. N, normal mice; Veh., vehicle-administered and LPS-treated mice; NSR, nonheat-processed* Scutellariae* Radix-administered and LPS-treated mice; HSR, heat-processed* Scutellariae* Radix-administered and LPS-treated mice. Data are the mean ± SEM. Significance: ^*∗*^ *p* < 0.05, ^*∗∗*^ *p* < 0.01, and ^*∗∗∗*^ *p* < 0.001 versus vehicle-administered and LPS-treated mice.

The protein levels of the oxidative stress-related proteins, such as I*κ*B*α*, I*κ*B*β*, and NF-*κ*Bp65, were examined. In the vehicle-administered and LPS-treated mice, the protein expression of I*κ*B*α* was significantly reduced and that of I*κ*B*β* was slightly reduced compared with the normal group (Figures 4(a) and 4(b)). Nonheat-processed* Scutellariae* Radix administration showed no difference compared with vehicle-administered acute lung injury mice, and heat-processed* Scutellariae* Radix led to a significant upregulation of I*κ*B*α* protein expression. Moreover, the vehicle-administered and LPS-treated mice showed upregulation of the nuclear NF-*κ*Bp65 protein compared to normal mice. On the other hand, the administration of nonheat-processed and heat-processed* Scutellariae* Radix led to a significant downregulation of NF-*κ*Bp65 protein expression (Figure 4(c)).

### 3.7. Inflammation-Related Protein Expressions in the Lung

**Figure 5:**
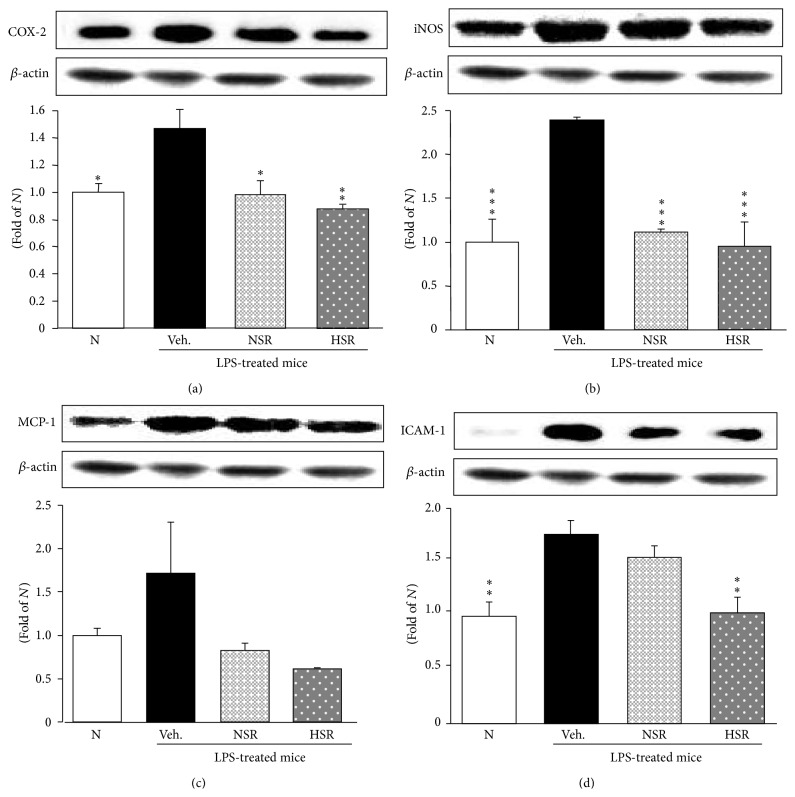
COX-2 (a), iNOS (b), MCP-1 (c), and ICAM-1 (d) protein expressions in lung tissue. N, normal mice; Veh., vehicle-administered and LPS-treated mice; NSR, nonheat-processed* Scutellariae* Radix-administered and LPS-treated mice; HSR, heat-processed* Scutellariae* Radix-administered and LPS-treated mice. Data are the mean ± SEM. Significance: ^*∗*^ *p* < 0.05, ^*∗∗*^ *p* < 0.01, and ^*∗∗∗*^ *p* < 0.001 versus vehicle-administered and LPS-treated mice.

**Figure 6:**
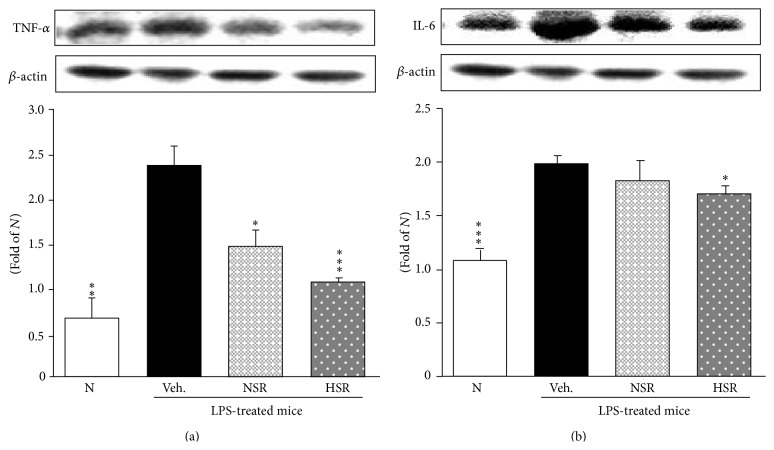
TNF-*α* (a) and IL-6 (b) protein expressions in lung tissue. N, normal mice; Veh., vehicle-administered and LPS-treated mice; NSR, nonheat-processed* Scutellariae* Radix-administered and LPS-treated mice; HSR, heat-processed* Scutellariae* Radix-administered and LPS-treated mice. Data are the mean ± SEM. Significance: ^*∗*^ *p* < 0.05, ^*∗∗*^ *p* < 0.01, and ^*∗∗∗*^ *p* < 0.001 versus vehicle-administered and LPS-treated mice.

Next, we quantified COX-2, iNOS, MCP-1, ICAM-1, TNF-*α*, and IL-6 protein expressions (Figures 5 and 6). The inflammation-related protein expressions in the vehicle-administered and LPS-treated mice were significantly augmented in the lung compared with normal mice. However, treatment with nonheat-processed and heat-processed* Scutellariae* Radix suppressed these proteins in the lung; especially, heat-processed* Scutellariae* Radix reduced these to nearly to normal levels in the lung (Figures 5(a)–5(d) and Figure 6(a)), except for IL-6 protein expression (Figure 6(b)).

## 4. Discussion

Herbal medicine has long been used for the treatment of diverse diseases. These include heat-dissipating Chinese herbs. Herbs in this group can reduce heat, purge fire, dry dampness, stop bleeding, and excretion of toxic material.* Scutellariae* Radix, a heat clearing herb, has antipyretic, hepatoprotective, antihypertensive, diuretic, and antibiotic activities [19].* Scutellariae* Radix is clinically used in two ways: nonheat-processed and heat-processed forms. Several studies have identified active flavonoids isolated from* Scutellariae* Radix: baicalin, baicalein, and wogonin [20, 21]. Flavonoids show medicinal and pharmacological activities against inflammation, allergy, viruses, and cancer [22, 23]. These three phenolic compounds were reported to have protective effects against LPS-induced acute lung injury in an animal model. However, there has been no comparative study on the effects of* Scutellariae* Radix according to heat treatment. The HPLC analysis indicated that the total components of heat-processed* Scutellariae* Radix showed a more than 24.9% increase compared to those of nonheat-processed* Scutellariae* Radix. In the present study, we demonstrated that heat-processed* Scutellariae* Radix was more effective than nonheat-processed* Scutellariae* Radix in an LPS-induced acute lung injury model. This suggests that heat-processed* Scutellariae* Radix exhibits marked potential as a therapeutic agent for acute lung injury.

LPS or endotoxin causes a marked inflammatory and immune response in the host. LPS injection results in acute lung injury, which is widely used in animal models to investigate the mechanisms of endotoxin-related acute lung injury. LPS-induced acute lung injury was characterized by the loss of alveolocapillary membrane integrity, leakage of plasma protein, pulmonary edema, marked neutrophil infiltration, and release of proinflammatory cytokines and mediators. In the present study, we evaluated the body weight change, lung weight, and histological change to quantify the magnitude of pulmonary edema and inflammatory cell infiltration, which is a typical symptom of inflammation and a major characteristic of acute lung injury [2]. The administration of nonheat-processed* Scutellariae* Radix significantly decreased the body weight change, lung weight, and histological change. However, heat-processed* Scutellariae* Radix alleviated the symptoms more than nonheat-processed* Scutellariae* Radix.

Oxidative stress plays an important role in the development of LPS-induced acute lung injury and is associated with ROS formation, where the excessive production of ROS leads to an imbalance of the antioxidant system and finally causes cell damage. LPS is well known as a potent stimulator of iNOS expression, which further causes the overproduction of NO [24]. The iNOS-induced excessive NO plays an important role by directly inducing tissue dysfunction and ONOO^−^ formation. Inhibitions of ROS, NO, and ONOO^−^ are major factors to alleviate acute lung injury [25]. In our experiment, the levels of ROS, NO, and ONOO^−^ in the lung were significantly reduced regardless of nonheat-processed* Scutellariae* Radix treatment. Moreover, the administration of heat-processed* Scutellariae* Radix was much lower than that of nonheat-processed* Scutellariae* Radix. Based on the results obtained in this study, because heat-processed* Scutellariae* Radix inhibited NO production itself, it may be more effective than nonheat-processed* Scutellariae* Radix.

Antioxidant activities involving scavenging ROS are mediated by antioxidant enzymes such as SOD and catalase. These enzymes stimulate the repair of cells or their resistance to damage caused by the accumulation of ROS due to environmental stresses [26]. As a matter of fact, SOD and catalase are considered to be the first line of defense against free radical attack. Increasing free radicals including O_2_^−^ generated by ROS accumulate in large amounts even inside cells. SOD is thoroughly utilized in scavenging O_2_^−^ via dismutation reaction. This reaction produces H_2_O_2_ as a byproduct, which is also a potentially reactive radical. Then, catalase neutralizes H_2_O_2_ to form H_2_O [27]. In this study, the oral administration of nonheat-processed* Scutellariae* Radix enhanced SOD and catalase activities. In particular, SOD and catalase activities on the administration of heat-processed* Scutellariae* Radix were increased significantly higher than with nonheat-processed* Scutellariae* Radix. This suggests that heat-processed* Scutellariae* Radix may effectively scavenge oxyradicals during the inflammatory response in the presence of LPS-induced acute lung injury.

NF-*κ*B is a transcriptional factor sequestered in the cytoplasm by the inhibitor protein I*κ*B*α*. Upon activation by LPS, I*κ*B*α* is rapidly phosphorylated and degraded, leading to the release of NF-*κ*Bp65. Then, NF-*κ*B translocates to the nucleus and promotes the transcription of target genes such as TNF-*α* and IL-6 [23, 28, 29]. We investigated the degradation of I*κ*B*α*, an inhibitor of NF-*κ*B, and NF-*κ*B activation. The results from the present study show that heat-processed* Scutellariae* Radix blocked the degradation of I*κ*B*α* and prevented the translocation NF-*κ*B in the lung. Namely, only heat-processed* Scutellariae* Radix markedly suppressed NF-*κ*B activation through the inhibition of I*κ*B*α* degradation. TNF-*α* and IL-6 appear in the early phase of the acute inflammatory response and play an important role in the pathophysiology of inflammation in acute lung injury [30, 31]. The elevated TNF-*α* and IL-6 protein expressions were significantly lowered with the administration of heat-processed* Scutellariae* Radix.

A previous study reported increases of pulmonary MCP-1 and ICAM-1 expressions in LPS-induced acute lung injury [32]. MCP-1 seems to play a primary role in many inflammatory states. It is expressed by various cells including monocytes/macrophages and controls recruitment [33]. With the administration of* Scutellariae* Radix, regardless of whether or not it received heat treatment, MCP-1 was reduced, but there was no significance. ICAM-1 protein is mainly located on the surface of endothelial cells. The level of expression of ICAM-1 on endothelial cells is increased following endothelial injury. The present experimental results show that the level of ICAM-1 protein expression was decreased on receiving heat-processed* Scutellariae* Radix.

The protein expressions of iNOS, which generates NO, and COX-2, which generates prostaglandin E2, contribute to the pathophysiological progression of acute lung injury [34]. LPS-induced increases in iNOS and COX-2 expressions in the lung are regulated by NF-*κ*B activation. Nonheat-processed and heat-processed* Scutellariae* Radix pretreatment markedly suppress expressions of iNOS and COX-2 after LPS injection in the lung.

## 5. Conclusion

**Figure 7:**
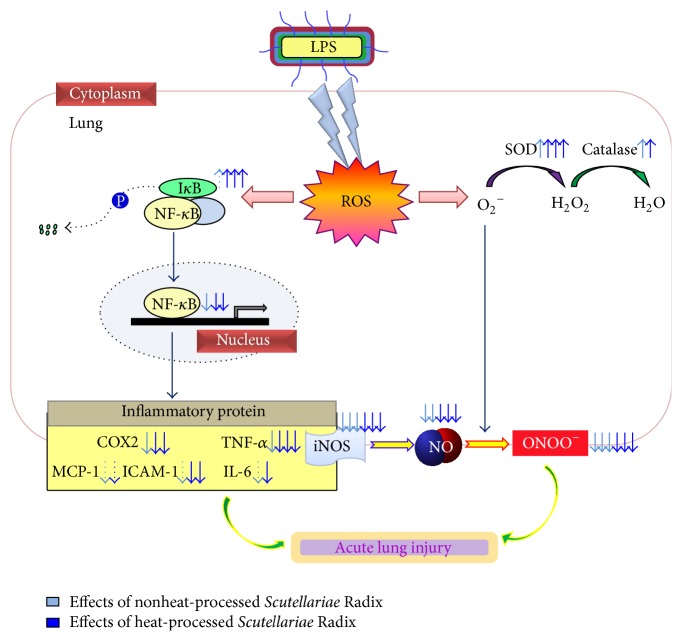
Predicted mechanisms in lung tissue on nonheat-processed or heat-processed* Scutellariae* Radix administration.

Heat-processed* Scutellariae* Radix leads to an increase in the contents of major flavonoids such as baicalin, baicalein, and wogonin. Heat-processed* Scutellariae* Radix has a protective effect against acute lung injury and exhibits stronger anti-inflammatory activity through the elevation of antioxidant enzymes and reduction of I*κ*B*α* degradation and NF-*κ*B activity compared to those of nonheat-processed* Scutellariae* Radix. This study suggests that heat processing of* Scutellariae* Radix may promote its lung-protecting potential through the inhibition of oxidative stress-sensitive mechanisms of the proinflammatory response, as shown in Figure 7.

## Figures and Tables

**Table 1:** Body weight gain and lung weight.

Group	Body weight			Lung weight (mg/g body weight)
	Initial (g)	Final (g)	Gain (g/3 days)	
Normal mice	29.6 ± 0.4	31.5 ± 0.5^*∗∗∗*^	1.8 ± 0.2^*∗∗∗*^	7.4 ± 0.5^*∗∗∗*^
LPS-treated mice				
Veh.	29.6 ± 0.4	25.2 ± 0.3	−4.3 ± 0.3	15.3 ± 0.8
NSR	29.6 ± 0.4	26.7 ± 0.5^*∗*^	−3.4 ± 0.2^*∗*^	10.3 ± 1.1^*∗∗*^
HSR	29.6 ± 0.4	26.4 ± 0.4^*∗*^	−3.3 ± 0.2^*∗*^	8.2 ± 0.4^*∗∗∗*^

Veh., vehicle-administered and LPS-treated mice; NSR, nonheat-processed *Scutellariae* Radix-administered and LPS-treated mice; HSR, heat-processed *Scutellariae* Radix-administered and LPS-treated mice. Data are the mean ± SEM. Significance: ^*∗*^ *p* < 0.05, ^*∗∗*^ *p* < 0.01, and ^*∗∗∗*^ *p* < 0.001 versus vehicle-administered and LPS-treated mice.

**Table 2:** Hematological analyses.

Group	MCP-1 (pg/mL)	IL-6 (pg/mL)	RANTES (pg/mL)	ROS (fluorescence/min/mL)	NO_2_^−^/NO_3_^−^ (*μ*mol/mL)	ONOO^−^ (fluorescence/mL)
Normal	0.4 ± 0.2^*∗∗∗*^	5.3 ± 1.4^*∗∗∗*^	4.8 ± 0.7^*∗∗∗*^	42.7 ± 3.4^*∗∗∗*^	15.9 ± 2.4^*∗∗∗*^	28.9 ± 0.5^*∗∗∗*^
LPS-treated mice						
Veh.	494.9 ± 44.3	284.9 ± 33.8	370.4 ± 71.9	72.7 ± 2.4	191.6 ± 9.3	35.2 ± 0.5
NSR	331.1 ± 36.8^*∗*^	218.2 ± 34.6	314.1 ± 32.7	62.8 ± 4.0	163.2 ± 8.9	32.1 ± 0.6^*∗∗*^
HSR	253.8 ± 84.2^*∗*^	163.5 ± 42.7^*∗*^	277.6 ± 38.8	58.8 ± 2.2^*∗∗*^	99.8 ± 32.9^*∗∗*^	30.7 ± 0.8^*∗∗*^

Veh., vehicle-administered and LPS-treated mice; NSR, nonheat-processed *Scutellariae* Radix-administered and LPS-treated mice; HSR, heat-processed *Scutellariae* Radix-administered and LPS-treated mice. Data are the mean ± SEM. Significance: ^*∗*^ *p* < 0.05, ^*∗∗*^ *p* < 0.01, and ^*∗∗∗*^ *p* < 0.001 versus vehicle-administered and LPS-treated mice.

**Table 3:** Oxidative stress biomarkers in lung tissue.

Group	ROS (fluorescence/min/mg protein)	NO (mM/mL)	ONOO^−^ (fluorescence/mg protein)
Normal mice	88 ± 3^*∗∗∗*^	0.96 ± 0.17^*∗∗*^	1,075 ± 51^*∗∗∗*^
LPS-treated mice			
Veh.	147 ± 11	1.87 ± 0.19	2,300 ± 84
NSR	132 ± 5	1.20 ± 0.10^*∗∗*^	1,430 ± 36^*∗∗∗*^
HSR	122 ± 6^*∗*^	0.63 ± 0.04^*∗∗∗*^	1,317 ± 47^*∗∗∗*^

Veh., vehicle-administered and LPS-treated mice; NSR, nonheat-processed *Scutellariae* Radix-administered and LPS-treated mice; HSR, heat-processed *Scutellariae* Radix-administered and LPS-treated mice. Data are the mean ± SEM. Significance: ^*∗*^ *p* < 0.05, ^*∗∗*^ *p* < 0.01, and ^*∗∗∗*^ *p* < 0.001 versus vehicle-administered and LPS-treated mice.
